# Obesity and Metabolic Syndrome in Childhood Leukemia and in Long-Term Survivors: Causes and Personalized Treatments

**DOI:** 10.3390/cancers17213446

**Published:** 2025-10-27

**Authors:** Francisco José Corominas-Herrero, Diana Navas-Carrillo, Juan Antonio Ortega-García, Isabel Martínez-Romera, Esteban Orenes-Piñero

**Affiliations:** 1 Department of Biochemistry and Molecular Biology-A, Universidad de Murcia, 30100 Murcia, Spain; franciscojose.corominas@um.es; 2 Department of Surgery, Hospital Clínico Universitario Virgen de la Arrixaca (HCUVA), 30120 Murcia, Spain; dnc45h@ad.sms.carm.es; 3 Paediatric Environmental Health Specialty Unit, Department of Pediatrics, Hospital Clínico Universitario Virgen de la Arrixaca (HCUVA), 30120 Murcia, Spain; ortega@pehsu.org; 4 Pediatric Haematology and Oncology Unit, Hospital Universitario La Paz, 28046 Madrid, Spain; imromera@salud.madrid.org

**Keywords:** childhood leukemia, long-term survivors, nutrition, obesity, metabolic syndrome

## Abstract

**Simple Summary:**

Acute lymphoblastic leukemia (ALL) is the most common pediatric cancer, accounting for about 34% of all cases. Survival rates have significantly improved to around 85% due to advances in chemotherapy, radiotherapy, and supportive care. However, new challenges have emerged, particularly the rising prevalence of obesity and metabolic syndrome among survivors. This review examines the link between leukemia treatment, obesity, and metabolic risk. Key factors such as cranial radiotherapy, corticosteroid use, and younger age at diagnosis are associated with increased weight gain and long-term metabolic issues. Genetic factors like FTO, MC4R, and LEPR polymorphisms may also contribute. Survivors often have poor dietary habits, including low micronutrient intake and high-fat, calorie-dense diets. Obesity and metabolic syndrome are linked to higher cardiovascular morbidity and reduced quality of life. Personalized medicine approaches that integrate genomics, metabolomics, and lifestyle data show promise for targeted prevention and intervention strategies.

**Abstract:**

Acute lymphoblastic leukemia (ALL) remains the most frequent pediatric malignancy, accounting for approximately 34% of all pediatric cancers, with remarkable improvements in survival (approximately 85%) due to advances in chemotherapy, radiotherapy, and supportive care. However, as survival rates have increased, new challenges have emerged—particularly the growing prevalence of obesity and metabolic syndrome among survivors. This review compiles evidence from the past decade on the relationship between leukemia treatment, obesity, and metabolic risk. The findings indicate that cranial radiotherapy, corticosteroid use, and younger age at diagnosis are key risk factors for excessive weight gain and long-term metabolic disturbances. Genetic factors such as FTO, MC4R, and LEPR polymorphisms may further influence susceptibility to obesity. Nutritional analyses highlight poor diet quality, insufficient micronutrient intake, and high-fat, energy-dense dietary patterns in survivors. Beyond endocrine dysfunction, obesity and metabolic syndrome are associated with elevated cardiovascular morbidity and reduced quality of life. Personalized medicine approaches—integrating genomics, metabolomics, and lifestyle data—hold promise for targeted prevention and intervention strategies. Early detection, continuous metabolic monitoring, and health education remain essential components in the long-term management of childhood leukemia survivors. In this review, we analyzed the dietary patterns of children and long-term leukemia survivors explaining why higher rates of obesity and comorbidities appear during or after treatments, and discussed interventions to prevent these conditions.

## 1. Introduction

Acute lymphoblastic leukemia (ALL) is the most frequently diagnosed pediatric cancer and it primarily affects children between the ages of 2 and 5, although it can occur at any age [1]. Advances in treatment have significantly improved survival rates for children with ALL, which now exceed 85%, with some high-risk subgroups receiving specialized therapies.

The second most common pediatric leukemia is acute myeloid leukemia (AML) and typically affects older children and adolescents [2]. The prognosis for AML can vary widely, with survival rates generally lower than for ALL. However, intensive chemotherapy and stem cell transplantation have improved outcomes for certain patients. Ranked third in the hematological malignancies, Hodgkin’s lymphoma is one of the most treatable cancers in children. The survival rate for children with Hodgkin’s lymphoma exceeds 90%, particularly when chemotherapy and radiation therapy are used.

ALL can be defined as a predominantly pediatric disease, as children comprise 80% of ALL cases, whereas in AML, children represent only about 10% of cases [2]. Importantly, leukemia, currently contributes up to one-third of all newly diagnosed childhood cancers each year and at least one chronic medical condition has been reported in about 50% of childhood leukemia survivors in their 20 s [3]. Leukemias are a heterogeneous group of diseases characterized by a replacement of normal bone marrow and blood elements with immature cells (blasts) and the accumulation of these cells in other tissues. Hematopoiesis is abnormal even before the proportion of cells in the marrow shows a perceptible increase. The precursors of immature leucocytes exhibit malfunctioning and progressively replace bone marrow and infiltrate other tissues [4].

The occurrence of childhood ALL is approximately 3 to 4 cases per 100,000 children under 15 years old, and it varies across different racial and ethnic groups. The highest rates are observed among Hispanic children, while African American children tend to have the lowest incidence [5]. Although ALL can affect children of any age, its peak incidence is typically between two and five years old, with a slight male predominance [6]. The various subtypes of ALL exhibit differences in their biological, cellular, and molecular features, as well as in their responses to treatment and the likelihood of relapse [6]. Due to advances in therapy and supportive care, the 5-year survival rate for pediatric ALL now exceeds 85% [5]. This progress has been driven largely by personalized treatment approaches incorporating pharmacodynamics and pharmacogenomics, risk-adapted therapies, and better supportive measures. Nevertheless, more than 80% of children worldwide reside in low-resource settings, where cure rates often do not exceed 35% [7]. Notably, in developing countries, most deaths occur early in treatment, mainly from infections and chemotherapy-related complications [7].

On the other hand, AML is a clonal disorder of hematopoietic tissue characterized by the uncontrolled proliferation of myeloid progenitor cells [8]. This results in the inadequate production of mature, healthy blood cells, which are often malignant. Advances in AML prognosis have been achieved through disease stratification into risk groups based on cytogenetic analysis, early response to treatment assessment, and the detection of chemotherapy induction failure. Currently, the chance of curing AML in developed countries is approximately 60% [9].

Previous studies have shown that obesity is a late-onset side effect among childhood cancer survivors (CCS), particularly in specific groups such as those who have survived brain tumors and leukemia [10]. It is crucial to highlight that therapies targeting the central nervous system (CNS)—such as cranial irradiation and/or intrathecal chemotherapy—can cause direct damage to the hypothalamic-pituitary region, although cranial irradiation has been substituted with time and is no longer part of the treatment for more than 95% of the patients. The damage of the hypothalamic-pituitary region can disrupt hormonal signaling pathways responsible for regulating hunger, appetite, and body fat balance, including hormones like ghrelin and leptin. These hormonal alterations may influence food consumption and appetite regulation, leading to increased food cravings [10]. While chronic heart conditions and early cardiac-related deaths in leukemia survivors have been associated with childhood treatment exposures, recent evidence suggests that having one or multiple cardiovascular risk factors—including obesity and other elements of metabolic syndrome (MS)—can increase this risk by up to 40-fold [11]. Early detection of high-risk groups, combined with a management plan that incorporates metabolic and nutritional evaluations and promotes increased physical activity, is vital in the treatment of childhood leukemia patients [11].

## 2. Materials and Methods

**Figure 1:**
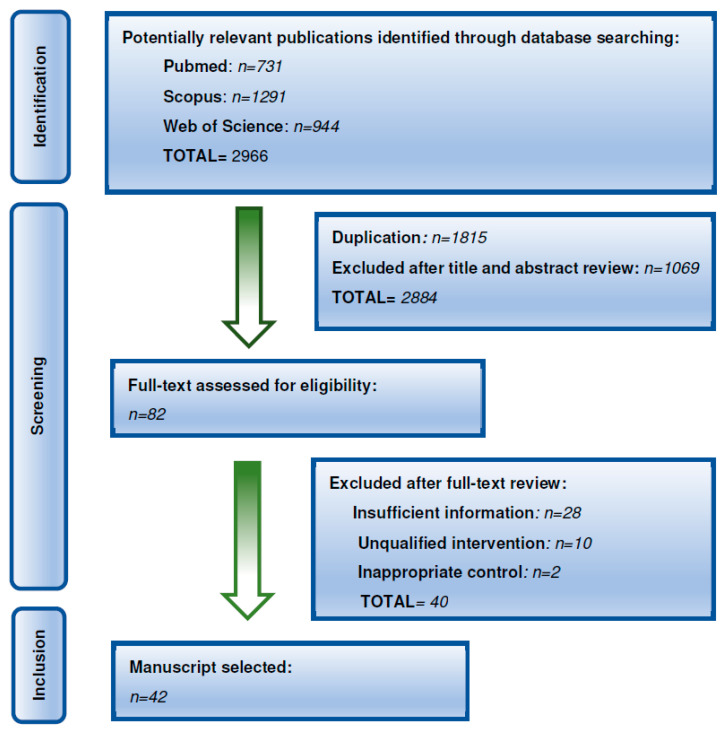
Flow diagram summarizing the screening process.

Published data for this narrative review were identified through searches of PubMed, Scopus and Web of Science databases. The search strategy for the results included articles published in the last ten years, so as to compile the most up-to-date evidence related to leukemia and nutrition (Figure 1). The search was limited systematic reviews, randomized clinical trials, clinical trials, meta-analysis and articles with high evidence to ensure that the review provided an accurate picture of current research. For that, the Scottish Intercollegiate Guidelines Network (SIGN) was followed and only studies with a level of scientific evidence of 1++, 1+, 2++ were included. The inclusion criteria were: articles in Spanish and English and articles published in the last 10 years. The exclusion criteria were: articles duplicated, articles with insufficient information, articles with low evidence (lower than 1++, 1+ and 2++), articles describing unqualified intervention, and articles with inappropriate control. The search and selection were performed by two independent authors and in case of differences, the articles were reviewed for all authors and a decision was made in common. A two-step approach was employed. First, the incidence and effects of leukemia in childhood were explored using the keywords “childhood leukemia”, “acute lymphoblastic leukemia”, “acute myeloid leukemia”, and “incidence of leukemia in children”. Then, the association between nutrition and leukemia was investigated using the keywords “nutrition in children with leukemia”, “obesity in children with leukemia”, “childhood cancer survivors” and “leukemia, obesity and treatments”. Bibliographies of all selected articles and references provided within them were screened to identify additional relevant studies.

## 3. Obesity and MS in Leukemia Survivors

### Obesity in Leukemia Survivors

Obesity is characterized by an abnormal or excessive accumulation of body fat, which the World Health Organization defines as a body mass index (BMI) of 30 kg/m^2^ or higher. BMI (weight/height^2^; kg/m^2^) is used as an indirect measure of body fatness in children and adolescents and should be compared with population growth references adjusted for sex and age [12]. However, abdominal or central obesity has been associated with increased cardiometabolic risk in children and adolescents. For waist circumference there are regional and international growth references allowing adjustment for age and sex. A waist-to-height ratio of more than 0.5 is increasingly used as an indicator of abdominal adiposity in clinical and research studies, with no need for a comparison reference [12]. Among adult survivors of childhood acute lymphoblastic leukemia (ALL), the prevalence of obesity has been reported to range from 11% to 56%, depending on the methods used to assess BMI and the characteristics of the studied populations (Table 1). Data from the North American Childhood Cancer Survivor Study, which calculated BMI based on self-reported height and weight, revealed that approximately 17% of ALL survivors (*n* = 1765), with a mean age of 24.1 years (range 18–42), had a BMI ≥ 30 kg/m^2^ [13]. As in the general population, obesity prevalence among ALL survivors increases with age. Longitudinal studies further show that obesity prevalence rises over time, with about 31.7% of survivors reaching BMI levels indicative of obesity by age 32 [13]. Notably, survivors who received cranial radiation therapy (CRT) had a significantly greater increase in BMI than those who received chemotherapy alone, which did not substantially affect BMI levels [13]. Moreover, younger age at CRT exposure was identified as a significant modifier of risk.

Weight gain in children treated for ALL often begins during therapy, persists after treatment cessation, and is associated with younger age at diagnosis [14]. Corticosteroids, such as prednisone and dexamethasone, play a central role in ALL treatment, particularly during induction and consolidation phases, and are key contributors to therapy-related weight gain [4]. While these agents promote leukemic cell apoptosis and have anti-inflammatory effects, they are also linked to metabolic side effects, notably increased appetite, fluid retention, altered glucose metabolism, and fat redistribution [15]. These effects can compromise both physical and psychological health, emphasizing the need for careful monitoring and supportive care during therapy.

Data from the Children’s Oncology Group and the Children’s Cancer Group, involving 1638 children with high-risk ALL, showed that 23% had BMI ≥ 95 th percentile at the end of treatment, compared to 14% at diagnosis [16]. Similarly, two other studies observed higher obesity rates at the end of therapy versus diagnosis (23% vs. 19% and 21% vs. 11%, respectively; *p* < 0.001) [17,18]. Obesity rates remained relatively stable five years post-treatment, with the highest prevalence observed in children diagnosed between ages 3 and 5 [19].

Nutritional status significantly influences chemotherapy tolerance and overall survival [20]. Lifestyle factors, such as maladaptive eating habits, reduced physical activity, and lower energy expenditure, also contribute to overweight and obesity in childhood cancer survivors (CCS) [21]. CCS exhibit higher rates of overweight/obesity than peers, along with increased risk of cardiopulmonary and metabolic complications [21]. Notably, CCS face elevated risks of chronic conditions, including hypertension, dyslipidemia, insulin resistance, diabetes, and early-onset obesity [22]. The first report linking BMI to treatment-related mortality during intensive pediatric AML chemotherapy found that underweight and overweight patients had significantly lower survival and higher treatment-related mortality than middleweight patients, with overweight children at the highest risk [23].

Primary risk factors for obesity after childhood ALL treatment include CRT, younger age at diagnosis, and female sex. A study found that females receiving CRT doses > 22 Gy had the highest likelihood of developing obesity (OR 3.81, 95% CI 2.34–5.99; *p* < 0.001) [24]. CRT exposure often led to anterior hypopituitarism, with deficiencies in growth hormone (GH) and luteinizing/follicle-stimulating hormones (LH/FSH). Importantly, untreated GHD was significantly associated with decreased muscle mass and exercise tolerance; untreated LH/FSHD was associated with hypertension, dyslipidemia, low BMD, and slow walking; and both deficits, independently, were associated with abdominal obesity, low energy expenditure, and muscle weakness [24].

In a cohort of 114 young adult ALL survivors (52 males, 62 females), those who underwent CRT showed lower IGF-1 levels, higher total, abdominal, and visceral fat, and elevated leptin concentrations compared to non-CRT survivors [25]. These findings suggest that CRT increases adiposity, reduces fat-free mass, elevates metabolic risk, and alters IGF-1 and leptin signaling [25]. Another study including eighty-two patients with a median age of 13.2 years confirmed higher leptin levels among CRT-exposed overweight survivors, with soluble leptin receptor levels inversely correlated with overweight status [26]. A significant increase in leptin levels was also found in overweight patients compared to the non-overweight patients. Interestingly, the prevalence of overweight in that cohort was higher than in the general European population (31% vs. 20%). Additionally, a systematic review of 863 ALL children and adolescents reported lower ghrelin levels than healthy controls, potentially due to inflammation and hyperlipidemia, with CRT-related hypothalamic dysfunction further impairing metabolic regulation [27]. Leptin, besides regulating appetite, promotes proinflammatory cytokines and may contribute to cardiovascular risk, highlighting its role as a potential marker for high-risk survivors [27].

CRT has been progressively replaced by intrathecal chemotherapy due to long-term adverse effects [7]. This shift has reduced risks of obesity and metabolic syndrome while maintaining CNS disease control, although corticosteroids and other systemic therapies still contribute to long-term metabolic risks [28,29].

Genetic factors may also influence obesity in ALL survivors. It is elsewhere accepted that radiation at a young age may affect the hypothalamus causing leptin receptor insensitivity and, therefore, a polymorphism in the leptin receptor (LEPR) gene, Gln223Arg, might influence susceptibility to obesity in survivors of childhood ALL. A study genotyped 600 non-Hispanic white adult ALL survivors enrolled onto the Childhood Cancer Survivor Study, and showed that among females treated with > or = 20 Gy cranial radiation, Arg/Arg individuals had six times higher odds of having BMI > or = 25 kg/m^2^ than those with a Gln allele (*p* = 0.04) [30], showing that LEPR polymorphism may influence obesity in female survivors of childhood ALL, particularly those exposed to cranial radiation. On the other hand, another study investigated the relationship between the rs9939609T > A polymorphism of the FTO gene in a cohort of 191 ALL survivors aged 4–26 years [31], showing that among patients treated with CRT, those homozygous for the rs9939609T allele had a significantly lower incidence of overweight after treatment compared to before treatment. These results suggest that the rs9939609T allele may protect against overweight in CRT-treated patients. In another study, a GWAS conducted on 1996 adult CCS (median age at diagnosis, 7.2 years; median age at follow-up, 32.4 years) identified potential obesity-associated loci on chromosomes 13 (FAM155A), 2 (SOX11), 4 (GLRA3), and 5 (CDH18, BASP1), particularly among CRT-exposed survivors [32]. Interestingly, genetic variants related to neural connectivity may modify the risk of obesity among survivors who receive CRT. These findings support the idea that CRT-induced neuronal damage may modulate obesity risk via genetic and epigenetic mechanisms [32]. A meta-analysis of 1458 adult ALL survivors confirmed two novel loci associated with BMI (LINC00856 rs575792008 and EMR1 rs62123082) [33]. It was observed that although adult survivors of childhood ALL have genetic heritability for BMI similar to that observed in the general population, treatment with CRT can modify the effect of genetic variants on adult BMI in childhood ALL survivors [33].

Beyond CRT, treatment-related factors linked to adult obesity include corticosteroid dose, therapy-related insulin resistance or diabetes, and weight gain during therapy [32]. Even in non-CRT patients, corticosteroid use and reduced physical activity contribute to increased body fat, elevated leptin, and long-term obesity risk [32,34]. These findings underscore the importance of early preventive strategies to mitigate lasting metabolic consequences of cancer therapy [32,34].

**Table 1:** Summary of studies analyzing obesity in childhood leukemia survivors.

Reference	Methods and Patients	Main Findings
[13]	Data from the North American Childhood Cancer Survivor Study. 1765 ALL survivors and 2167 siblings.	Higher rates of obesity among ALL survivors were associated with increasing age. ALL survivors who were treated with CRT had a significantly greater increase in BMI and younger age at CRT exposure significantly modified risk.
[16]	Data from Children’s Oncology Group and the Children’s Cancer Group: 1635 Children with ALL.	Prevalence of obesity is significantly lower at diagnosis than at the end of therapy (14 vs. 23%).
[17]	183 children with ALL.	Prevalence of obesity is significantly lower at diagnosis (19%) than at the end of therapy (23%).
[18]	165 children with ALL.	11% of obese patients at diagnosis compared with 21% at the end of therapy.
[23]	768 AML children from the Children’s Cancer Group-2961.	Overweight AML children were significantly less likely to survive and more likely to experience treatment-related mortality than middle-weight patients.
[24]	748 participants treated with CRT from St Jude Lifetime Cohort study	ALL patients with CRT doses > 20 Gy presented anterior hypopituitarism with significant deficiencies in GH and LH/FSH.
[25]	114 young adult survivors of childhood ALL	Increased total body fat, abdominal fat, visceral fat, and serum leptin levels among survivors treated with CRT when compared to those not treated with CRT
[26]	ALL survivors who received CRT (*n* = 82) compared to those who did not (*n* = 116)	Increase in leptin levels and reduction in soluble leptin receptor in plasma overweight patients compared to the non-overweight patients.
[27]	Systematic review with 863 ALL children and adolescents	Levels of ghrelin in children with ALL were lower than in controls. Higher leptin serum levels were associated with body fatness in ALL survivors.
[30]	Childhood Cancer Survivor Study; *n* = 600 ALL patients.	LEPR Gln223Arg polymorphism may influence obesity in female survivors of childhood ALL, particularly those exposed to CRT.
[31]	191 ALL patients receiving CRT.	9939609T allele of the FTO gene could be a protecting factor against obesity as a negative association was found between this allele and overweight in ALL survivors who received CRT.
[32]	GWAS among 1996 adult CCS from the St. Jude Lifetime Cohort (median age at diagnosis, 7.2 years; median age at follow-up, 32.4 years).	Potential genetic predictors of obesity were found on chromosomes 13 (FAM155A), 2 (SOX11), 4 (GLRA3), and 5 (CDH18 and BASP1) among patients exposed to CRT. Most of them were associated with neuronal growth, repair, and connectivity.
[33]	1458 adult survivors of childhood ALL (median time from diagnosis, 20 years) from Childhood Cancer Survivor Study and St. Jude Lifetime Cohort Study.	Although adult survivors of childhood ALL have a similar genetic heritability for BMI to that observed in the general population, treatment with CRT could modify the effect of genetic variants on adult BMI.
[35]	Retrospective cohort of 183 pediatric ALL patients	During ALL therapy, patients are at risk for early development of elevated BMI and blood pressure, which places them at potentially increased risk for future adverse health conditions.
[36]	893 CCS with a mean follow-up of 14.9 years from Emma Children’s Hospital/Academic Medical Center	Increased prevalence of obesity in CCS. Risk factors for developing a high BMI at follow-up were a younger age, a high BMI at diagnosis and treatment with cranial radiotherapy.
[37]	3467 participating in the Childhood Cancer Survivor Study	Survivors treated with CRT or SRT exhibited a two- to threefold increased risk of adult short stature, hypothyroidism, and infertility. CRT was associated with an increased risk of being overweight/obese.
[38]	36 children and adolescents between 10–21 years old newly diagnosed ALL treated with intravenous methotrexate	Delayed elimination at 48 h of plasma methotrexate was associated with approximately 2-fold higher risk for larger size and greater obesity.

Abbreviations: ALL: Acute lymphoblastic leukemia; AML: Acute myeloid leukemia; BMI: Body mass index; CRT: Cranial radiation therapy; FTO: fat mass and obesity-associated gene; GH: Growth hormone; GWAS: Genome wide association study; LEPR: Leptin receptor gene; LH/FSH: Luteinizing hormone/follicle-stimulating hormone; SRT: Spinal radiotherapy.

## 4. Metabolic Syndrome in Leukemia Survivors

Excess body fat, commonly referred to as obesity, represents just one of several cardiovascular risk factors commonly observed in adults who survived childhood acute lymphoblastic leukemia (ALL). Metabolic syndrome (MS)—a cluster of risk factors for cardiovascular disease and type 2 diabetes—affects up to 33.6% of these survivors. According to the National Cholesterol Education Program Adult Treatment Panel III (NCEP-ATP III) criteria, MS is diagnosed if at least three of the following are present: (I) waist circumference exceeding 102 cm in men or 88 cm in women; (II) triglycerides over 150 mg/dL; (III) HDL cholesterol below 40 mg/dL; (IV) blood pressure of 130/85 mmHg or higher; and (V) fasting glucose of at least 100 mg/dL [39].

Similar to obesity, the prevalence of MS—or comparable cardiovascular risk profiles—tends to increase with age among ALL survivors, is more common in females than males, and has been associated with prior exposure to cranial radiotherapy (CRT) and/or growth hormone (GH) abnormalities [40] (Table 2).

To our knowledge, the earliest study examining cardiovascular risk after childhood ALL identified obesity and abnormal lipid profiles in a small cohort of survivors treated with CRT compared to those receiving chemotherapy alone [41]. Within this cohort of 26 individuals, 62% had at least one cardiovascular risk factor potentially linked to their cancer treatment, and 30% had more than two. Additionally, CRT-exposed individuals exhibited significantly higher body mass index (BMI) (*p* = 0.039), triglycerides (*p* = 0.027), and very low-density lipoprotein (VLDL) levels (*p* = 0.022) compared to the chemotherapy-only group [41], indicating an increased risk of MS in CRT-treated survivors.

A subsequent matched case–control study of 44 ALL survivors, assessed approximately 17 years post-treatment, investigated GH deficiency and cardiovascular risk. Participants treated with either CRT or chemotherapy had significantly higher levels of insulin (*p* = 0.002), glucose (*p* = 0.01), low-density lipoprotein (LDL) (*p* < 0.05), apolipoprotein B (*p* < 0.05), triglycerides (*p* < 0.05), and leptin (*p* < 0.05) than matched controls [42]. They also displayed higher BMI, larger waist-to-hip ratios, increased fat mass, and lower lean body mass (*p* < 0.001). Doppler echocardiography further revealed impaired cardiac function, including reduced ejection fraction (*p* < 0.001) and shortening fraction (*p* = 0.01). Notably, more than 90% of the cohort were GH deficient, suggesting that CRT-induced GH deficiency is a key contributor to MS development in this population [42].

More recently, a larger prospective multicenter study (*n* = 184) evaluated the prevalence of MS in young adults surviving childhood leukemia, with a mean follow-up of 15.4 years [43]. It was found that the overall prevalence of MS was 9.2%, with a significantly higher risk observed in the irradiated group in both univariate and multivariate analyses (*p* = 0.03). Irradiation was additionally associated with elevated triglycerides (OR = 4.5; *p* = 0.004), lower HDL (OR = 2.5; *p* = 0.02), and higher fasting glucose (OR = 6.1; *p* = 0.04). These results confirm previous findings in a single-center cohort of 500 adult survivors (228 females) with a median follow-up of 19 years, in which ALL survivors treated with cranial irradiation had an elevated risk of MS compared to those not receiving CRT (23% vs. 7%, *p* = 0.011), probably determined by higher prevalence of overweight and hypertension [44]. These observations show that adult CCS, especially those treated with cranial irradiation, are at increased risk of developing the metabolic syndrome.

A cross-sectional study assessed the relationship between body composition, metabolic profile, adipokines, and carotid intima-media thickness (cIMT) in 55 young ALL survivors [45]. Patients were divided into two groups based on CRT exposure (25 irradiated, 30 non-irradiated) and compared with 24 leukemia-free controls. CRT exposure affected visceral and subcutaneous adipose tissue accumulation (*p* < 0.05 for both), as measured by CT scan. In multiple linear regression, cIMT (a subclinical atherosclerosis marker) positively correlated with CRT exposure (*p* = 0.029), diastolic BP (*p* = 0.016), and leptin-to-adiponectin ratio (*p* = 0.048), confirming CRT’s impact on fat distribution and potential role in atherosclerosis. Besides, leptin-to-adiponectin ratio and diastolic BP, both MS-related factors, also influenced cIMT [45].

In a recent study of 53 ALL children, 3 were overweight at diagnosis, increasing to 13 by the end of the study (*p* = 0.04) [46]. Additionally, mean blood leptin levels were also higher in overweight patients than in non-overweight patients (*p* = 0.001). Moreover, MS was observed in 21 patients, with a significant increase in BMI Z-score over the study period (*p* = 0.001). All these findings underscore the rise of MS in ALL survivors’ post-treatment, highlighting the importance of nutritional interventions to prevent future cardiovascular diseases.

## 5. Dietary Patterns in Childhood Cancer Survivors

In an analysis conducted by the Healthy Living Study 2011–2012 from 67 eligible patients, 22 agreed to participate in the study, including 17 diagnosed with pediatric ALL and 5 diagnosed with lymphoma [47]. It was found that in the first 6 and 12 months of follow-up, patients reported that they had more preference for fast foods than for sweet foods (*p* = 0.03), carbohydrate meals (*p* = 0.0007), and fats (*p* < 0.0001).

A separate investigation analyzed dietary intake among 640 participants enrolled in the Dana-Farber Cancer Institute ALL Consortium Protocol. Using a food frequency questionnaire, researchers evaluated nutritional intake and compared it to the Dietary Reference Intake (DRI), stratifying results by ALL risk classification (standard risk [SR] and high risk [HR]). Findings revealed that, across all age groups, average calorie consumption exceeded recommended levels, with the most pronounced differences seen in younger children compared to adolescents [48]. No significant variation in total caloric intake was found between SR and HR groups. However, a striking result emerged regarding fat intake: a substantial proportion of male participants consumed fat outside the acceptable macronutrient distribution range (*p* < 0.001). Regarding micronutrients, there were no significant differences in the intake of vitamins C and E between the SR and HR groups. Yet, vitamin D intake showed a significant disparity among females, with those in the HR group consuming less than those in the SR group (*p* = 0.02), while the same was not true for males (*p* = 0.15). Importantly, for vitamin D, over 90% of participants in nearly all subgroups failed to meet the DRI. Regarding calcium, a greater percentage of children classified as HR were below the recommended intake, whereas a higher proportion of SR participants met the guidelines. This unequal distribution of calcium intake between risk groups was evident in both sexes (*p* = 0.03 for males and 0.02 for females) [48]. These results highlight specific dietary intake concerns that differ according to sex and ALL risk status, suggesting a need for targeted nutritional interventions in this population.

Conversely, another study evaluated the dietary patterns of 22 pediatric survivors of ALL and lymphoma (median age: 11.7 years) by comparing their intake with the 2010 Dietary Guidelines for Americans (DGA). The analysis found that factors such as race, gender, ethnicity, and treatment status did not significantly influence diet quality among these childhood cancer survivors (CCS) [49]. However, when individual dietary components were assessed, the participants showed low adherence to recommendations for whole fruits, total vegetables, dark green vegetables and legumes, seafood and plant-based proteins, healthy fatty acids, and sodium. Furthermore, none of the survivors met the fiber intake recommendations, and only 29% complied with the guideline of limiting saturated fat intake to below 10% of total energy. On average, fiber consumption reached just 63% of the recommended daily intake, while energy from saturated fat exceeded the recommended threshold by 15% [49].

In terms of micronutrients, none of the children met the recommended potassium intake, and only 24% achieved adequate calcium consumption. Notably, 81% of participants consumed sodium beyond the upper recommended limit. Vitamin intake was similarly inadequate: just 5% of the group met the RDI for vitamin D, 19% for both vitamin E and choline, and 29% for vitamin K [49]. A more recent investigation explored the link between vitamin D status and metabolic biomarkers in a well-characterized cohort of ALL survivors [50]. It revealed a significant positive association between vitamin D levels and plasma HDL-cholesterol concentrations in female participants, but not in males, suggesting a possible sex-specific cardioprotective role for vitamin D. This is particularly relevant given that ALL survivors often face multiple cardiovascular risk factors, underlining the critical need for effective dietary strategies to support the long-term health of this population.

In another study analyzing the effects of nutritional status prior to treatment with folate and vitamin B12 on chemo-induction performed in 46 children with ALL, hypoalbuminemia, vitamin B12, and folate deficiencies were all found to be significantly associated with treatment-related mortality during chemotherapy induction [51]. This study also observed the sequential changes in B12 and folate levels due to chemotherapy, finding that both folate and B12 levels decreased during follow-up. For instance, at baseline, the mean serum folate level was 8.5 pg/mL; after the first month, it declined to 7.2 pg/mL, and by the second month to 4.65 pg/mL (*p* = 0.001) [52]. Regarding patient outcomes, incomplete bone marrow recovery at day 14 of induction (*p* = 0.007), delayed peripheral blood count recovery (*p* = 0.001), and toxic induction deaths (*p* = 0.003) were more common among folate-deficient children compared with folate-replete ones [51].

In agreement with previous observations, a progressive decline in folate levels during intrathecal methotrexate chemotherapy has been documented. Notably, these levels decline as methotrexate dosage increases, leading to higher toxicity and potential reductions in cure rates [53,54]. Folate and vitamin B12 supplementation may help mitigate these toxicities in patients with deficiencies [53]. However, folate supplementation during cancer chemotherapy has been controversial, due to concerns that folates may reduce chemotherapy efficacy and potentially support neoplastic cell growth [54]. These findings warrant confirmation through similar studies with larger sample sizes from diverse regions, particularly in resource-limited areas with higher prevalence of folate and B12 deficiencies. Importantly, several studies in adults receiving treatment for malignant pleural mesothelioma also report improved median overall survival in patients supplemented with folic acid and vitamin B12 [52,55].

On the other hand, maternal intake of micronutrients may significantly impact the risk of childhood leukemia through several biologically plausible pathways. This relationship is supported by a substantial body of research highlighting the role of one-carbon metabolism nutrients in genetic and epigenetic processes critical to fetal development, as well as the significance of maternal nutritional status in shaping the child’s immune system [56,57].

A study focused on the period before leukemia treatment investigated maternal prenatal consumption of individual one-carbon metabolism nutrients. The findings suggested that higher maternal intake of these nutrients—both from diet and supplements—prior to conception was associated with a lower risk of developing acute lymphoblastic leukemia (ALL), and potentially acute myeloid leukemia (AML) as well [58]. While most research to date has focused on maternal vitamin supplements, fewer studies have explored the effects of dietary sources of folate and other one-carbon metabolism nutrients. One case–control study, which included 333 leukemia cases and 695 frequency-matched controls, observed that increased dietary intake of folate and vitamin B12 during the last six months of pregnancy correlated with a reduced risk of ALL. Interestingly, however, higher intake of vitamin B6 from food was unexpectedly linked to an elevated risk of the disease. Notably, the strongest associations with ALL were found among mothers who reported alcohol consumption during pregnancy [59].

Altogether, these findings indicate that survivors of pediatric ALL, especially long-term survivors, tend to exhibit poor diet quality. Evidence is also accumulating that many of these survivors experience unhealthy weight gain early in therapy, with a substantial proportion becoming overweight or obese after treatment ends.

## 6. Weight Gain and Effect of Therapy in Childhood Leukemia

Excessive weight gain occurring during treatment for ALL is usually related to the use of corticosteroids, alterations in appetite regulation, radiotherapy to the CNS, and reduced energy expenditure due to physical inactivity. This excess weight has been widely documented in ALL survivors [60]. This way, in a retrospective cohort of 183 pediatric ALL patients diagnosed from 2000 to 2008, the influence of anti-neoplastic treatment in critically ill children with cancer was analyzed [35]. it was found that, at diagnosis, 36% of the patients were overweight and 19% were classified as obese. Towards the end of treatment, 49% of the patients were overweight and 21% were obese, respectively [35].

Weight gain following treatment has been strongly linked to CRT, with female survivors being disproportionately affected, as demonstrated in a study of 1765 adult survivors of childhood cancer participating in the Childhood Cancer Survivor Study [13]. In that cohort, women who received > 20 Gy of CRT had a two- to threefold higher risk of obesity compared to their male siblings. Furthermore, individuals treated with CRT before the age of five were nearly four times more likely to be obese than their sibling controls [13]. Supporting the role of CRT in post-treatment weight gain, another study involving 893 CCS with a mean follow-up of 14.9 years was conducted [36]. For girls, an increased prevalence of obesity was found. Risk factors for developing a high BMI at follow-up were a younger age, a high BMI at diagnosis and treatment with cranial radiotherapy [36].

The distinct impacts of CRT, spinal radiotherapy (SRT), and total body irradiation (TBI) on growth and endocrine outcomes were also evaluated collectively in a cohort of 3467 of ALL and myeloid leukemia survivors from the Childhood Cancer Survivor Study [37]. Compared to those who had not received radiotherapy, survivors treated with CRT or SRT exhibited a two- to threefold increased risk of adult short stature, hypothyroidism, and infertility, defined as failure to achieve pregnancy or live birth. In contrast, exposure to TBI carried a five- to tenfold higher risk for these outcomes, and individuals treated with both CRT and TBI faced more than a tenfold increase in risk. With respect to alterations in body composition, CRT was the only radiotherapy modality significantly associated with an elevated risk of being overweight or obese (OR 1.6, 95% CI 1.3–1.9).

Interestingly, another study performed a prospective study examining body composition of 36 children and adolescents between 10–21 years old newly diagnosed ALL treated with intravenous methotrexate [38]. Body fat percentage was measured using dual-energy x-ray absorptiometry. All patients received 5 g/m^2^ of intravenous methotrexate infused over 24 h. Plasma methotrexate concentrations were measured at 24, 42, and 48 h to assess the elimination rate. Interestingly, it was found that delayed elimination at 48 h was associated with approximately 2-fold higher risk for larger size and greater obesity, thus confirming the importance of chemotherapy in body composition in LAA patients.

Another important issue is the relationship between BMI and the toxicity of ALL treatments. Considerable research has been conducted in patients with ALL with the aim of correlating BMI and outcomes in patients undergoing chemotherapy for hematological diseases [61]. Several studies described poorer survival rates in children with ALL and higher BMI. One study included 1443 children aged 2.0–17.9 years [61]. Patients were classified according to sex- and age-adjusted international childhood cut-off values, corresponding to adult body mass index: underweight, <17 kg/m^2^; healthy weight, 17 to <25 kg/m^2^; overweight, 25 to <30 kg/m^2^; and obese, ≥30 kg/m^2^. Obese children had a higher incidence rate ratio (IRR) for severe toxic events, liver and kidney failures, bleeding, abdominal complication, suspected unexpected severe adverse reactions and hyperlipidaemia compared with healthy-weight children. In addition, obese children aged ≥10 years had increased IRRs for asparaginase-related toxicities compared with healthy-weight older children, such as thromboses and anaphylactic reactions. In another study, elevated BMI was associated with increased toxicity, non-relapse mortality, and decreased overall survival among adolescents and young adults treated on Dana-Farber Cancer Institute consortium, and the deleterious effect of increased BMI was more pronounced in older adolescents and young adults CCS [62]. Interestingly, in CCS who were overweight/obese, worse outcomes were seen in older ones (4-year OS, 55% vs. 73%, *p* = 0.023). Regarding toxicity, CCS who were overweight/obese experienced higher rates of grade 3/4 hepatotoxicity and hyperglycemia (60.7% vs. 42.2%, *p* = 0.0005, and 36.4% vs. 24.4%, *p* = 0.014, respectively). In a previous study, the association between obesity and poorer event-free survival (EFS) in pediatric acute lymphoblastic leukemia was analyzed in 198 children between the ages of 1 and 21 years diagnosed with ALL between January 2008 and January 2013 at Children’s Hospital Los Angeles [63]. Importantly, 30 children (15.2%) were overweight and 41 (20.7%) were obese at diagnosis. Independent of established predictors of treatment response, obesity during induction was associated with significantly greater risk for persistent minimal residual disease (odds ratio, 2.57; 95% CI, 1.19 to 5.54; *p* = 0.016). Moreover, obesity and overweight were independently associated with poorer EFS (*p* = 0.012) [63].

On the other hand, therapy-related toxicities, whether acute or chronic, can impact treatment efficacy, overall survival (OS), and the patient’s quality of life. The six most important acute toxicities of ALL therapy are: venous thromboembolism, osteonecrosis, neurological sequelae, delayed methotrexate elimination, asparaginase-associated pancreatitis, and toxicities resulting from the new biological therapies [64]. Most of these severe acute toxicities of ALL treatment can be mitigated through tailored therapy adaptations for individual patients and careful incorporation of immunotherapy. These adaptations should become a central component of contemporary pediatric ALL protocols as soon as possible, and ultimately improve patients’ OS and wellness. Understanding more about the impact of BMI in pediatric leukemia treatments is of utmost importance to provide prompt intervention and improve outcomes.

## 7. Personalized and Precision Interventions to Prevent Obesity and MS

Historically, survival from childhood cancer was extremely low, with only one in every thousand children surviving a century ago. However, remarkable improvements in survival rates have been achieved since then. For this reason, it is essential to develop methods to optimize recovery and minimize side effects. Current treatments must aim for complete recovery while limiting adverse effects. The identification of oncogenes or tumor suppressor genes, as in many cancers, opens the path for molecular diagnosis and may even enable genetic counseling.

Recent advances in personalized and precision medicine are transforming the approach to obesity and metabolic syndrome in childhood leukemia survivors, a particularly vulnerable group due to the long-term effects of oncological treatments. These strategies rely on integrating genomic, epigenomic, metabolomic, and environmental data to design tailored and effective interventions. A key approach involves identifying genetic variants associated with predisposition to obesity and metabolic disturbances in these patients. Recent studies have highlighted that polymorphisms in genes such as FTO and the Melanocortin-4 Receptor gene (MC4R) influence susceptibility to post-treatment obesity, enabling risk stratification and personalized intervention planning [65]. Additionally, the analysis of biomarkers—such as adipokines, inflammatory cytokines, and metabolic hormones—facilitates early detection of metabolic dysfunctions, even before clinical symptoms appear, allowing for timely preventive measures.

Importantly, in leukemia survivors, genetic variants in the FTO and MC4R genes, known for their roles in obesity and appetite regulation, affect weight and metabolic health. Specifically, FTO polymorphisms have been linked to increased obesity risk and altered eating behaviors, while MC4R variants can influence appetite and satiety [31]. Understanding these genetic factors can help tailor interventions for weight management and overall health in this population. For instance, the FTO gene is strongly associated with obesity risk. Certain FTO gene variants, like the rs9939609 SNP, have been shown to increase the likelihood of developing obesity, particularly in individuals of European descent [65]. These variants may affect appetite, satiety, and food preferences, leading to increased calorie intake and weight gain, whereas the MC4R gene plays a crucial role in regulating appetite and energy balance. Variations in MC4R can impact an individual’s sensitivity to hunger and fullness signals, potentially leading to overeating or difficulty with weight loss [65].

It is critical to note that leukemia survivors, especially those treated with chemotherapy or radiation, are at an increased risk for weight gain and metabolic complications. The interplay of FTO and MC4R variants with other factors, such as treatment side effects and lifestyle choices, can significantly influence their metabolic health. Consequently, identifying specific FTO and MC4R variants in survivors could inform personalized interventions aimed at promoting healthy weight and preventing metabolic disorders [65]. For example, individuals with high-risk FTO genotypes might benefit from more intensive dietary and exercise interventions.

Regarding pharmacological therapies, targeted treatments that modulate metabolic pathways altered by chemotherapy and radiotherapy are currently under investigation. For example, GLP-1 receptor agonists, which are used in adults with obesity, are being studied in pediatric populations with treatment-resistant obesity, showing promising results in weight reduction and metabolic profile improvement [66].

Lifestyle interventions are also increasingly personalized through digital health technologies, enabling real-time monitoring and adaptation of diet and exercise programs to optimize adherence and effectiveness. Furthermore, advances in imaging techniques and metabolic assessments allow for more precise and early monitoring of components of metabolic syndrome, facilitating risk stratification and targeted interventions in survivors at higher risk of long-term complications.

Finally, integrating multi-omics data with clinical and environmental profiles is paving the way toward fully personalized medicine, where treatments are tailored not only to genetic characteristics but also to lifestyle and environmental exposures. Conducting clinical trials to validate these approaches will be essential for establishing standardized protocols and improving long-term outcomes in this vulnerable population.

A recent discovery has highlighted a connection between acute lymphoblastic leukemia (ALL) and the trans-activation response DNA-binding protein (TARDBP) [67]. TARDBP is an RNA/DNA-binding protein that contributes to the progression and metastasis of various cancers through activation of the β-catenin signaling pathway. In experimental models, TARDBP expression was manipulated in ALL cell lines using lentiviral transduction to either suppress or enhance its activity. In vitro results showed that silencing TARDBP led to reduced proliferation and cell cycle progression, along with increased apoptosis. In contrast, overexpression of TARDBP promoted cell growth and survival. Treatment with XAV-939, a β-catenin pathway inhibitor, effectively counteracted the aggressive characteristics induced by TARDBP overexpression in ALL cells. In vivo experiments further supported these findings: when TARDBP-silenced or TARDBP-overexpressing ALL cells were injected into mice, TARDBP stimulated leukemic cell expansion in the spleen and bone marrow. These results suggest that TARDBP plays a tumor-promoting role in ALL, and ongoing research may determine whether targeting this pathway directly could be a viable therapeutic strategy.

## 8. Limitations

There are some limitations in relation to this work. First, there is a limitation in the language as only works in English and in Spanish has been selected. This way, some important information could be missed. Second, there is substantial heterogeneity in the specific definition of obesity as it has changed with time. Thus, the heterogeneity of the anthropometric parameters used in the different studies, as well as the clinical outcomes evaluated, hindered performing a meta-analysis or showing pooled results. This highlights the importance of performing further studies in this field and our observations should be interpreted with caution at this time.

## 9. Conclusions

As in the general population, several factors contribute to weight gain in ALL survivors, as obesity is a multi-factorial condition. These factors include poor nutrition, physical inactivity, and individual genetic characteristics. Additionally, exposure to radiotherapy can alter body composition by increasing adiposity, reducing lean body mass, and enhancing metabolic complications.

Obesity remains a widespread and persistent global health issue. Its pervasive impact on society and cancer-related clinical research has elevated it to a priority research area for organizations such as the National Cancer Institute and the American Society of Clinical Oncology. Despite some conflicting findings, evidence increasingly supports a significant adverse effect of overweight and obesity on survival outcomes in pediatric leukemia. To better understand the influence of obesity on the treatment outcomes of acute lymphoblastic leukemia (ALL), additional prospective studies are warranted. Health professionals should prioritize early identification of patients at heightened risk and implement targeted intervention strategies—such as nutritional guidance—to reduce or prevent obesity, a common long-term sequela of cancer therapy.

To sum up, it is of paramount importance physician counselling for health promotion in cancer survivors, including encouraging a healthy diet, physical exercise, and avoidance of high-risk behaviors. Looking forward, epidemiology, genetics, or predictive modeling are expected to aid in predicting survivors at increased risk of developing obesity and cardiometabolic complications, as well as in tailoring treatments to minimize therapy-related toxicities.

## Figures and Tables

**Table 2:** Summary of studies analyzing metabolic syndrome in childhood leukemia survivors.

Reference	Methods and Patients	Main Findings
[41]	26 survivors of childhood ALL.	62% had at least one CVRF related to their treatment, and 30% had more than two. Subjects who were treated with CRT had an increased BMI (*p* = 0.039), triglyceride (*p* = 0.027) and VLDL levels (*p* = 0.022) when compared with those who received only chemotherapy. Survivors of childhood ALL treated with CRT were at risk for MS.
[42]	Matched case–control study with 44 ALL survivors treated with CRT or chemotherapy at a median of 17 years after treatment.	Significantly higher levels of insulin (*p* = 0.002), blood glucose (*p* = 0.01), LDL (*p* < 0.05), apolipoprotein B (*p* < 0.05), triglycerides (*p* < 0.05), and leptin (*p* < 0.05) were found among ALL patients compared with controls. ALL patients had higher BMI, waist to hip ratio, higher fat mass and lower lean mass (*p* < 0.001). Impaired indicators of cardiac function (ejection fraction; *p* < 0.001, shortening fraction; *p* = 0.01) were also observed among survivors. More than 90% of patients were GH deficient.
[43]	Prospective multicentric study (*n* = 184), evaluating the prevalence of MS in young adults surviving childhood leukemia with a mean follow-up of 15.4 years	Overall prevalence of MS was 9.2% with a significant higher risk observed in the group treated with TBI in the univariate and in the multivariate analysis. TBI was also associated with higher blood levels of triglycerides (OR = 4.5; *p* = 0.004), low levels of HDL (OR = 2.5; *p* = 0.02), and elevated fasting glucose (OR = 6.1; *p* = 0.04).
[44]	500 adult CCS, with a median follow-up time of 19 years	ALL survivors treated with cranial irradiation had an increased risk of developing MS compared with ALL survivors not treated with cranial irradiation (23% vs. 7%, *p* = 0.011)
[45]	Cross-sectional study in 55 young ALL survivors (25 irradiated and 30 non-irradiated) and 24 leukemia-free controls	Treatment with CRT had a significant effect on visceral adipose tissue and subcutaneous adipose tissue accumulation. cIMT positively correlated with exposure to CRT (*p* = 0.029), diastolic blood pressure (*p* = 0.016), and leptin-to-adiponectin ratio (*p* = 0.048). CRT modified the distribution of fat and could play a critical role in atherosclerosis. Leptin-to-adiponectin ratio and diastolic blood pressure, both associated with MS, also influenced cIMT.
[46]	53 ALL children	An increase of 20% of overweight patients was observed at the end of treatment (*p* = 0.04). Mean blood leptin level was also higher. BMI Z-score significantly increased over the study period (*p* = 0.001).

Abbreviations: ALL: Acute lymphoblastic leukemia; BMI: Body mass index; CCS: Childhood cancer survivors; cIMT: Carotid intima-media thickness; CRT: Cranial radiation therapy; CVRF: Cardiovascular risk factor; GH: Growth hormone; HDL: high-density lipoprotein; MS: Metabolic syndrome; OR: Odds-ratio; TBI: Total body irradiation; VLDL: Very low-density lipoprotein.

## Data Availability

The original contributions presented in this study are included in the article. Further inquiries can be directed to the corresponding author.

## Abbreviations

The following abbreviations are used in this manuscript:

ALL	Acute lymphoblastic leukemia
AML	Acute myeloid leukemia
BMI	Body mass index
CCS	childhood cancer survivors
CNS	Central nervous system
CRT	cranial radiation therapy
DRI	Dietary Reference Intake
EFS	Event-free survival
FTO	Fat mass and obesity-associated gene
GWAS	Genome wide association study
LEPR	Leptin receptor gene
MC4R	Melanocortin-4 Receptor gene
MS	Metabolic syndrome
OS	Overall survival
RDI	Recommended daily intake
TARDBP	Trans-activation response DNA binding protein
